# Zinc pyrithione activates K^+^ channels and hyperpolarizes the membrane of rat pulmonary artery smooth muscle cells

**DOI:** 10.1371/journal.pone.0192699

**Published:** 2018-02-23

**Authors:** Basma G. Eid, Alison M. Gurney

**Affiliations:** Faculty of Biology, Medicine and Health, University of Manchester, Manchester, United Kingdom; Newcastle University, AUSTRALIA

## Abstract

The membrane potential helps determine pulmonary artery smooth muscle cell (PASMC) contraction. The Kv7 channel activators, retigabine and flupirtine, are thought to dilate pulmonary arteries by hyperpolarising PASMC. Zinc pyrithione activates Kv7 channels by a mechanism distinct from retigabine and with different Kv7 subunit selectivity. This study aimed to determine if zinc pyrithione selectively activates Kv7 channels in rat PASMC to evoke pulmonary artery dilation. Zinc pyrithione relaxed pulmonary arteries with half-maximal effect at 4.3μM. At 10μM it activated pronounced voltage-dependent K^+^ current and hyperpolarized PASMCs by around 10mV. Tetraethylammonium ions (TEA, 10mM) and paxilline (1μM) abolished both the current and hyperpolarisation. XE991 (10μM) blocked the hyperpolarization and reduced the current by 30%. Iberiotoxin (50nM) had no effect on the hyperpolarisation, but reduced the current by 40%. The XE991-sensitive current activated with an exponential time course (time constant 17ms), whereas the iberiotoxin-sensitive current followed a bi-exponential time course (time constants 6 and 57ms), suggesting that the drugs blocked different components of the zinc pyrithione-induced current. Zinc pyrithione therefore appears to activate at least two types of K^+^ channel in PASMC; an XE991, TEA and paxilline-sensitive Kv7 channel and a TEA, paxilline and iberiotoxin-sensitive BK_Ca_ channel. Both could contribute to the relaxing effect of zinc pyrithione on pulmonary artery.

## Introduction

The membrane potential of pulmonary artery smooth muscle cells (PASMCs) regulates contraction. The resting potential depends on K^+^ ions leaking through K^+^ channels open at rest. Inhibitors of the resting K^+^ flux depolarise the membrane to open voltage-gated Ca^2+^ channels, resulting in Ca^2+^ entry and contraction. Opening additional K^+^ channels has the opposite effect, causing hyperpolarization, reduced Ca^2+^ entry and vasodilation [1,2]

PASMCs express many K^+^ channel genes, which yield multiple voltage-activated potassium channels, large conductance Ca^2+^-activated (BK_Ca_), ATP-sensitive (K_ATP_) and two pore-domain (K_2P_) K^+^ channels. The voltage-gated channels include members of the Kv7 family, encoded by the *KCNQ* genes. Pharmacological inhibition of Kv7 channels evokes pulmonary vasoconstriction while their activation with retigabine or flupirtine promotes vasodilation [3–5]. As dysfunctional Kv7 channels were implicated in the pathogenesis of hypertension, they have been suggested as a target for future anti-hypertensive drugs [6]. The main subunit indicated in the systemic circulation is Kv7.4, although it may function in a heteromeric complex with Kv7.5 [7,8]. Kv7.1 channels are also present and can be activated to evoke vasodilation, but don’t contribute in unstimulated arteries [5]. Pulmonary arteries express the *KCNQ1*, *KCNQ4* and *KCNQ5* genes [4], although the functional roles of the individual Kv7 subunits in PASMCs have still to be established. The relatively high expression of Kv7.4 suggests that it could be important [4,9] and reduced Kv7.4 expression is associated with the pathogenesis of pulmonary hypertension [10,11].

Zinc pyrithione (ZnPy) is a potent activator of Kv7 channels without efficacy at several voltage-gated K^+^ and Ca^2+^ channels [12]. Its structure differs markedly from retigabine and the two drugs consequently interact at distinct sites within the Kv7 channel and influence activity in different ways [13]. They also differ in subunit selectivity. Whereas retigabine activates all Kv7 isoforms except Kv7.1, ZnPy activates Kv7.1, Kv7.4 and Kv7.5 but not Kv7.3 [12]. These differences have proved helpful in the characterisation of Kv7 channel function in neurones [14,15]. Like retigabine, ZnPy activates K^+^ current in rat airways smooth muscle cells and relaxes intact airways [16]. Its effects on vascular muscle are, however, unknown, although ZnPy has been used to investigate zinc-dependent enzymes in pulmonary artery endothelial cells [17]. As the distinct properties of ZnPy could be useful for identifying Kv7 channel function in arteries, this study characterized its actions on isolated rat PASMCs. The aim was to determine if ZnPy selectively activates Kv7 channels in PASMCs to evoke membrane hyperpolarization and artery dilation.

## Materials and methods

### Tissue preparation

All work on animals was conducted with the approval of the Local Ethical Review Process of The University of Manchester and in accordance with the UK Scientific Procedures (Animals) Act 1986. Male Sprague-Dawley rats (250-300g) were killed by cervical dislocation as approved under schedule 1 of the act, describing appropriate methods of humane killing. Lungs were excised into physiological salt solution (PSS) containing (in mM): 120 NaCl, 5 KCl, 1 MgCl_2_, 0.5 NaH_2_PO_4_, 0.5 KH_2_PO_4_, 10 4-(2-hydroxyethyl)piperazine-1-ethanesulfonic acid (HEPES), 10 glucose and 1 CaCl_2_; pH 7.4 with NaOH. Intra-pulmonary arteries (300–600 μm) were cleaned of connective tissue and mounted in a small vessel myograph under 4mN of applied tension (Danish Myo Technology, Denmark). Vessels were bathed in PSS continually aerated at 37°C and left to recover for 30 min, washing every 15 min. A sustained level of tension was generated by exposing vessels to the α_1_-adrenoceptor agonist phenylephrine (1 μM) or XE991 (1–5 μM). The subsequent application of ZnPy (10 nM—100 μM) induced relaxation responses that were measured as the percentage of induced tone that remained.

### PASMC electrophysiology

Smooth muscle cells were isolated from pulmonary arteries and whole-cell patch clamp used to record membrane potential and K^+^ currents as described previously [18,19]. Patch pipettes were filled with solution of composition (in mM): 130 KCl, 1 MgCl_2_, 1 ethylene glycol-bis(2-aminoethylether)-N,N,N',N'-tetraacetic acid (EGTA), 20 HEPES and 0.5 Na_2_GTP; pH 7.3 with KOH. Junction potentials (<3mV) were not corrected. Membrane potential, under current clamp (at zero current), and K^+^ currents under voltage clamp were recorded using WinWCP software (University of Strathclyde) with a BNC-2090 digitizer (National instruments USA). Families of voltage-gated K^+^ currents were activated by 300ms steps from the holding potential of -80mV to between -70 and 60mV, applied at 5s intervals. Current amplitude was measured once it reached a plateau, as the average current between 250 and 265ms after the voltage step. To isolate non-inactivating current, cells were clamped at 0mV for ≥5 min as previously described (Evans *et al*. 1996) and the residual current at 0mV recorded. Unless otherwise stated, drugs were applied to cells from a pipette positioned >100μm away, using a gravity-fed perfusion system at ~2 ml/min. To avoid flow-induced artefacts, control solution was applied for at least 1min before switching to the drug.

### Drugs

Phenylephrine hydrochloride, ZnPy, glibenclamide and tetraethylammonium chloride (TEA) were from Sigma-Aldrich (Dorset, UK). Iberiotoxin, paxilline and XE991 (10,10-bis(4Pyridinylmethyl)-9(10H)-anthracenone) dihydrochloride were from Tocris (Bristol, UK). ZnPy (100mM), paxilline (10mM) and glibenclamide (10mM) were dissolved in dimethylsulphoxide, which was present at 0.01% when applying ZnPy, increasing to 0.02% or 0.11% when co-applied with paxilline or glibenclamide, respectively. Other drugs were dissolved in PSS or deionised water.

### Data analysis

Data are expressed as mean ± standard error of the mean (SEM), or mean with 95% confidence interval in brackets, of *n* cells. Analysis employed Excel spreadsheets and GraphPad Prism software (GraphPad Software, Inc., LaJolla, CA). Sample sizes vary due to the unpredictable nature of the patch-clamp technique: the essential “gigaseal” was often lost before protocols were completed. Data were tested for normality using the Shapiro-Wilk and Kolmogorov-Smirnov tests and parametric or non-parametric statistical tests employed as indicated. The test used in each case is specified next to the data and p<0.05 taken to indicate a significant effect. The maximum response of arteries to ZnPy and the concentration of ZnPy evoking half-maximal relaxation (IC_50_) were determined from least squares fits of the variable-slope Hill equation to the concentration-response curve (GraphPad Prism). The activation of voltage-gated current was fit with an exponential function of the form *Y*_*t*_ = *Y*_1_(1–*e*^−*t*/τ_1_^)+*Y*_2_(1–*e*^−*t*/τ_2_^), where *Y*_*t*_ is the current at time *t*, τ_*1*_ and τ_*2*_ are time constants and *Y*_*1*_ and *Y*_*2*_ the amplitudes of each of the two components. *Y*_*2*_ = 0 when fitting a single exponential function. Single and double exponential fits were compared using the extra sum-of-squares F-test and p<0.05 taken to indicate that 2 components gave a better fit.

## Results

**Fig 1:**
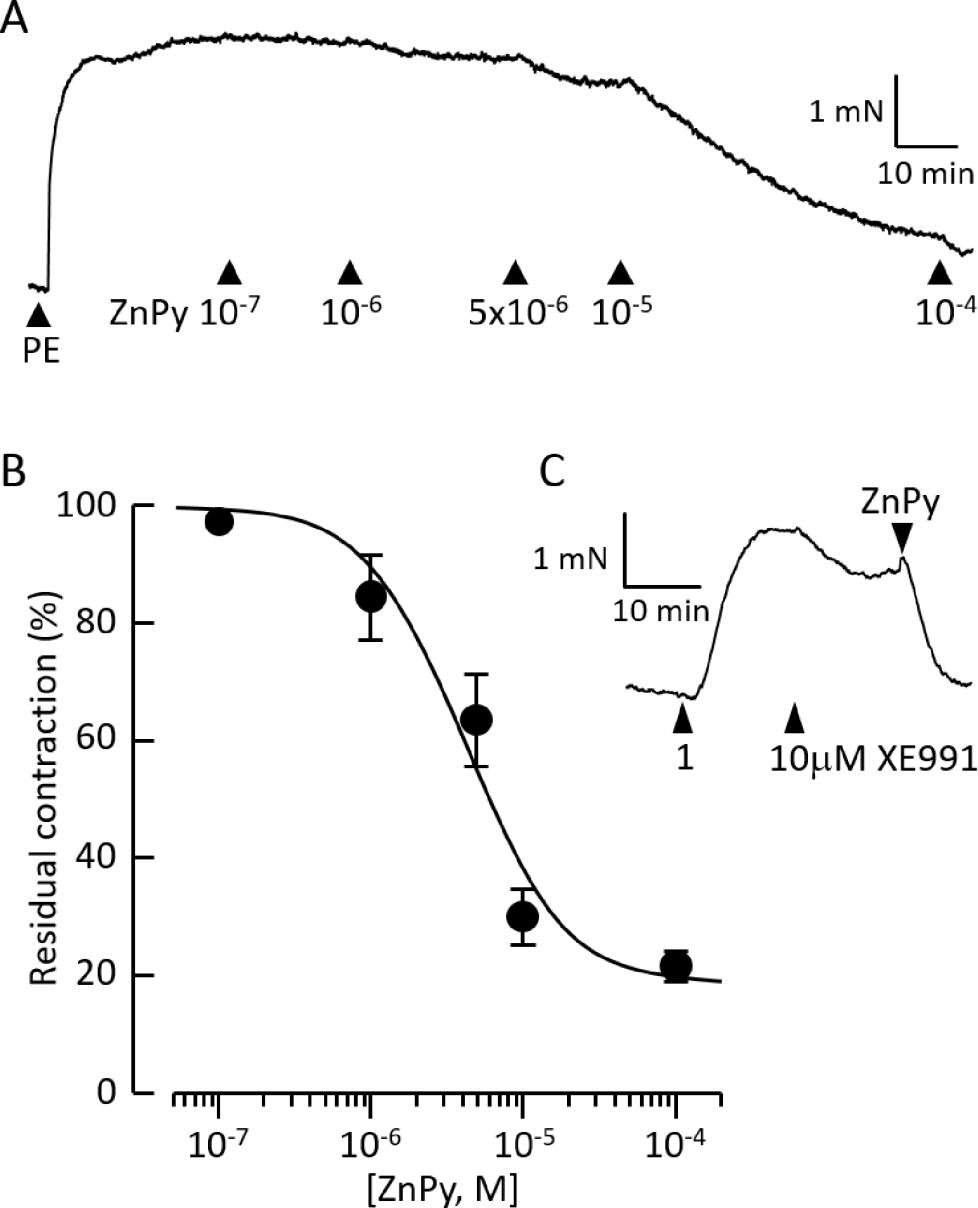
ZnPy relaxes pulmonary arteries. ***A*.** Record of tension developed in response to 1μM phenylephrine (PE), followed by cumulative addition of ZnPy (1x10^-7^ - 1x10^-4^ M). ***B*.** ZnPy concentration-response curve. Points and bars represent mean ± S.E.M. Curve is best fit to the Hill equation with IC_50_ = 4.3μM, minimum residual tension = 18.3% and Hill slope = 1.3. ***C*.** Record of tension developed in response to the cumulative addition of 1μM XE991, 10μM XE991 then 10μM ZnPy.

When applied to arteries constricted with phenylephrine (1 μM), ZnPy evoked concentration-dependent relaxation (Fig 1A and 1B) with IC_50_ = 4.3 ± 0.9 μM (n = 8). At the maximum response, seen above 10μM ZnPy, the phenylephrine-induced tension fell to 18 ± 6%. Time control arteries studied in parallel, but not exposed to ZnPy, showed no loss of tension over the same period. As reported previously [3], XE991 (1–10μM) evoked contraction comparable in amplitude to the phenylephrine response, with the maximum effect at 1μM (Fig 1C). Consequently, it was not possible to test the ability of XE991 to antagonise the effect of ZnPy on phenylephrine-constricted arteries. The constriction developed by XE991 was sustained for over an hour, but reversed immediately by 90±3% (n = 5) upon subsequent exposure to 10 μM ZnPy (Fig 1C).

### Zinc pyrithione evokes hyperpolarization

Isolated rat PASMCs had a mean resting membrane potential of -44±3 mV (n = 14), membrane capacitance of 18.1±0.8 pF (n = 116) and input resistance of 3.9±0.4 GΩ (n = 116).

**Fig 2:**
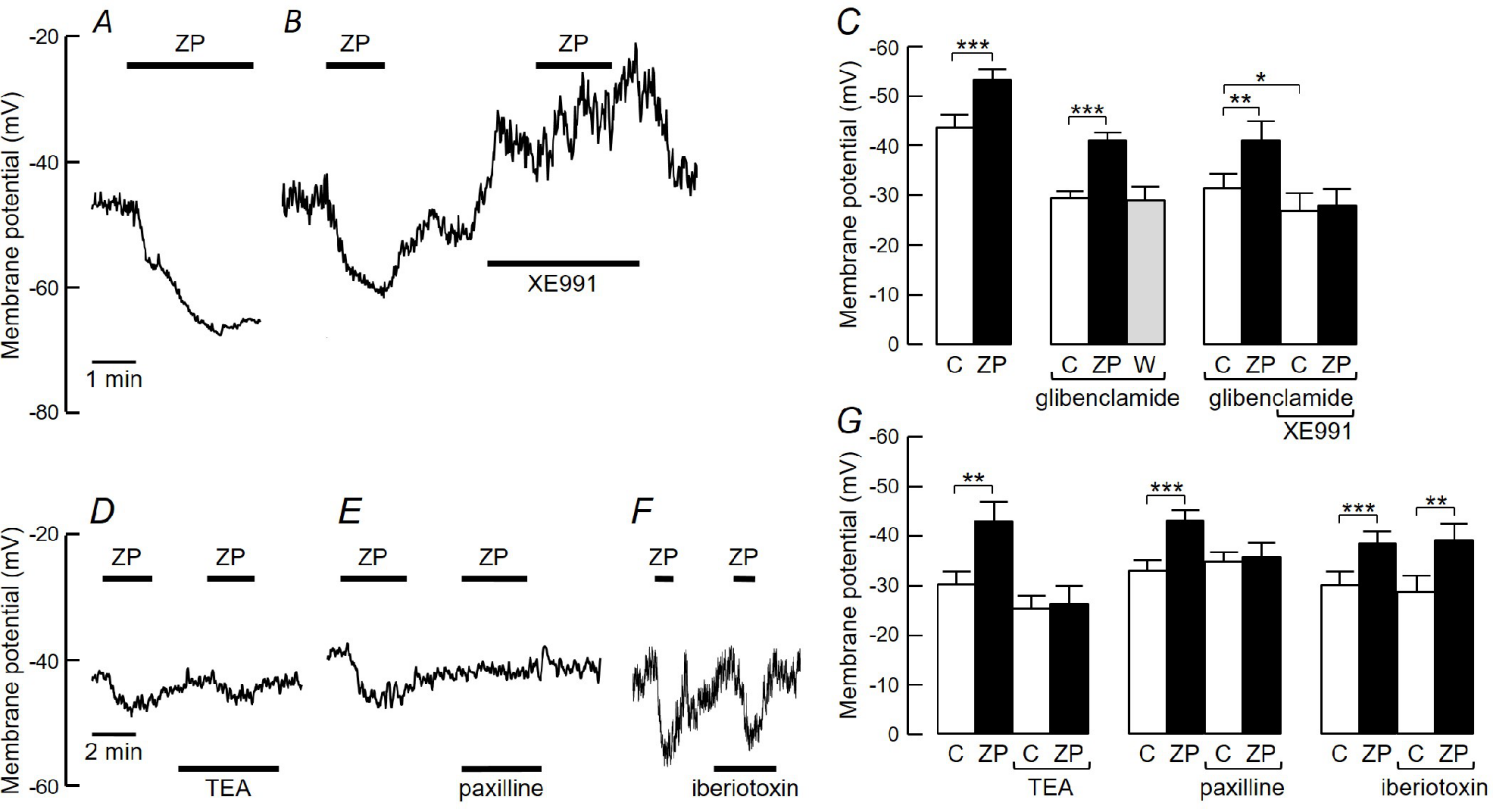
Properties of membrane hyperpolarisation induced by ZnPy in PASMC. ***A-B*** Original records of PASMC membrane potential before and after applying 10μM ZnPy (ZP) in the absence (***A***) and presence (***B***) of 10μM glibencamide, with and without the addition of 1μM XE991. ***C*.** Mean (±SEM) membrane potentials measured before and after applying ZnPy in the absence (n = 14) or presence (n = 12) of glibenclamide or glibenclamide plus XE991 (n = 12). C = control, W = wash. *p<0.05, **p<0.01, ***p<0.001 by repeated measures ANOVA with Tukey’s multiple comparisons test (glibenclamide present) or paired t-test (glibenclamide absent). ***D-F*.** Records of membrane potential during responses to ZnPy before and after exposing cells to 10mM TEA (***D***), 1μM paxilline (***E***) or 50nM iberiotoxin (***F***). ***G*.** Mean (±SEM) membrane potentials measured from the recordings in D-F. N = 10 (TEA), 15 (paxilline) and 12 (iberiotoxin). **p<0.01, ***p<0.001 by repeated measures ANOVA with Tukey’s multiple comparisons test.

ZnPy was applied to cells at 10μM as this is a submaximal concentration, but above the IC_50_ for artery relaxation and in the range of concentrations that activate Kv7 channels [13]. When applied after recording a stable membrane potential for 1–2 min, ZnPy evoked hyperpolarization that was significantly different from zero (Fig 2A and 2C) of 9±1.5 mV (n = 14, one sample t-test), although two of the cells did not respond. Since K_ATP_ channels can contribute to resting potential in PASMCs and can be pharmacologically activated [1,20], the effect of inhibiting K_ATP_ channels with glibenclamide was tested on the response to ZnPy. In the presence of 10μM glibenclamide, the resting potential was more depolarized than controls, but the addition of 10 μM ZnPy still caused hyperpolarization (Fig 2B and 2C) of 11±1.2 mV (n = 46; one sample t-test), comparable with hyperpolarization in the absence of glibenclamide. Upon repeated application, the ZnPy-induced hyperpolarisation was reproducible, with two applications spaced a few minutes apart changing the membrane potential by -8 ± 2 mV and -11 ± 3 mV (n = 17), respectively. Five out of the 46 cells did not hyperpolarize. Both in the absence and presence of glibenclamide, the effects of ZnPy were observed within a few seconds, took around 2 min to reach peak and recovered completely within 12 min after washing. Glibenclamide was present throughout the remaining experiments.

The Kv7 blocking drug, XE991 (10μM), caused significant depolarisation of PASMCs by 6±1.5 mV (n = 10, one-sample t-test), as previously reported [4]. When applied in the presence of XE991 and glibenclamide, ZnPy (10μM) did not significantly change the membrane potential (Fig 2B and 2C). Kv7 channels are inhibited by TEA (Hadley *et al*. 2000). When applied on its own, TEA (10mM) had no effect on the membrane potential, as previously reported [19], but it inhibited the effect of ZnPy, which in its presence did not evoke significant hyperpolarization (Fig 2D and 2G). As TEA is also a blocker of BK_Ca_ channels, the selective BK_Ca_ inhibitors, paxilline and iberiotoxin, were tested to determine any BK_Ca_ contribution to the ZnPy-induced hyperpolarization. Paxilline (1μM) had no effect on membrane potential, but it prevented ZnPy from evoking hyperpolarization (Fig 2E and 2G). Iberiotoxin (50 nM) also had no effect on membrane potential and it failed to inhibit the effect of ZnPy (Fig 2F and 2G), which in its presence still caused hyperpolarization of 10±2.5 mV (n = 12).

### Zinc pyrithione activates voltage-activated K^+^ current

**Fig 3:**
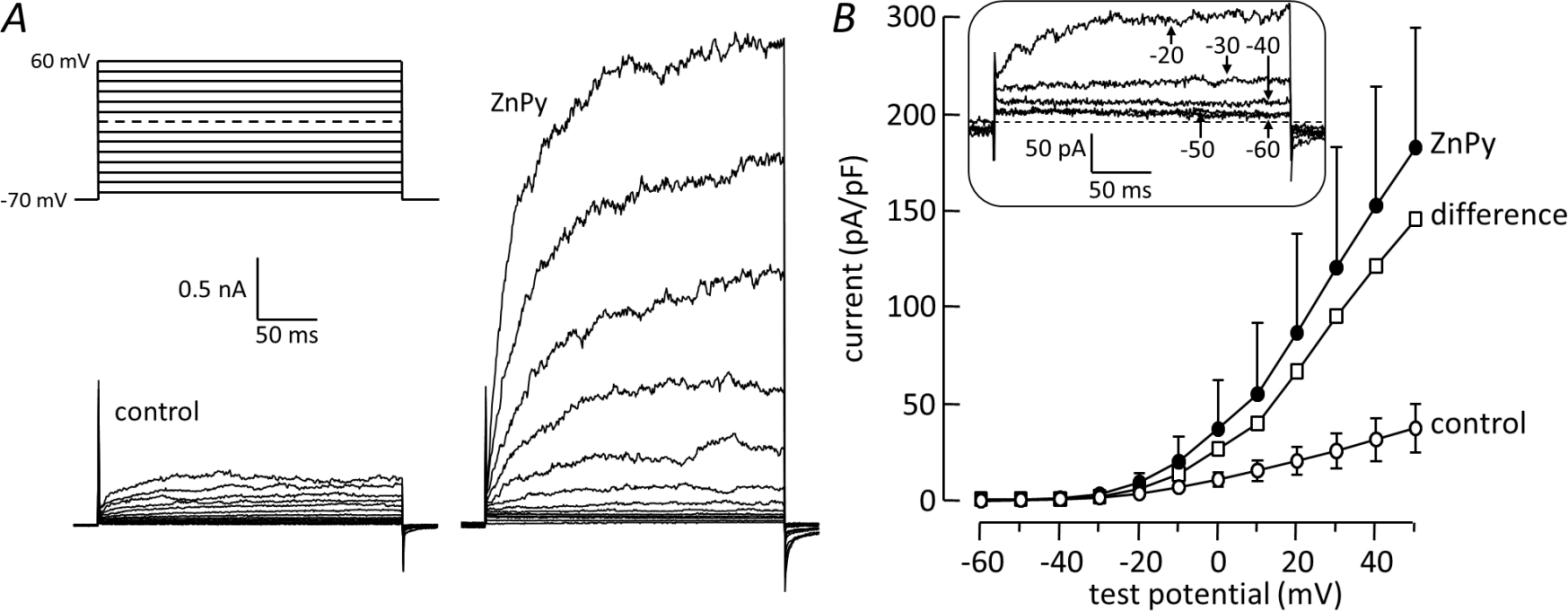
ZnPy potentiates voltage-gated K^+^ current in PASMCs. ***A*.** K^+^ currents activated by 300ms steps to between -70 and 60 mV, applied from a holding potential of -80 mV at 5s intervals. Records under control conditions (left) and in the presence of 10μM ZnPy (right). Voltage protocol above control records. ***B*.** Current density plotted as a function of test potential before (control) and after applying 10μM ZnPy; mean±SEM of 3 cells. The difference between measurements in control conditions and after exposure to ZnPy is shown to indicate the currents induced by ZnPy. Inset shows the averaged difference currents at test potentials from -60 to -20 mV, obtained by digitally subtracting the control records from those during exposure to ZnPy. Broken line indicates zero current.

Depolarizing voltage steps from -80 mV evoked outward currents that reached maximum within 50ms and increased in amplitude with depolarization (Fig 3A). ZnPy (10μM) had a profound effect on these currents, sometimes increasing the amplitude 10-fold as in Fig 3A. Fig 3B shows a plot of current density versus voltage. An increase in current amplitude in the presence of ZnPy was apparent at potentials above -30mV and grew with depolarisation. Subtracting the control amplitudes from the amplitudes in the presence of ZnPy revealed that ZnPy activated a voltage-dependent current. To assess the threshold voltage for current activation, the ZnPy-induced currents at steps to between -60 and -20 mV were revealed by digitally subtracting control from ZnPy traces. The inset in Fig 3B shows the average of these records from three cells: the first sign of increased current is apparent between -50 and -40 mV.

**Fig 4:**
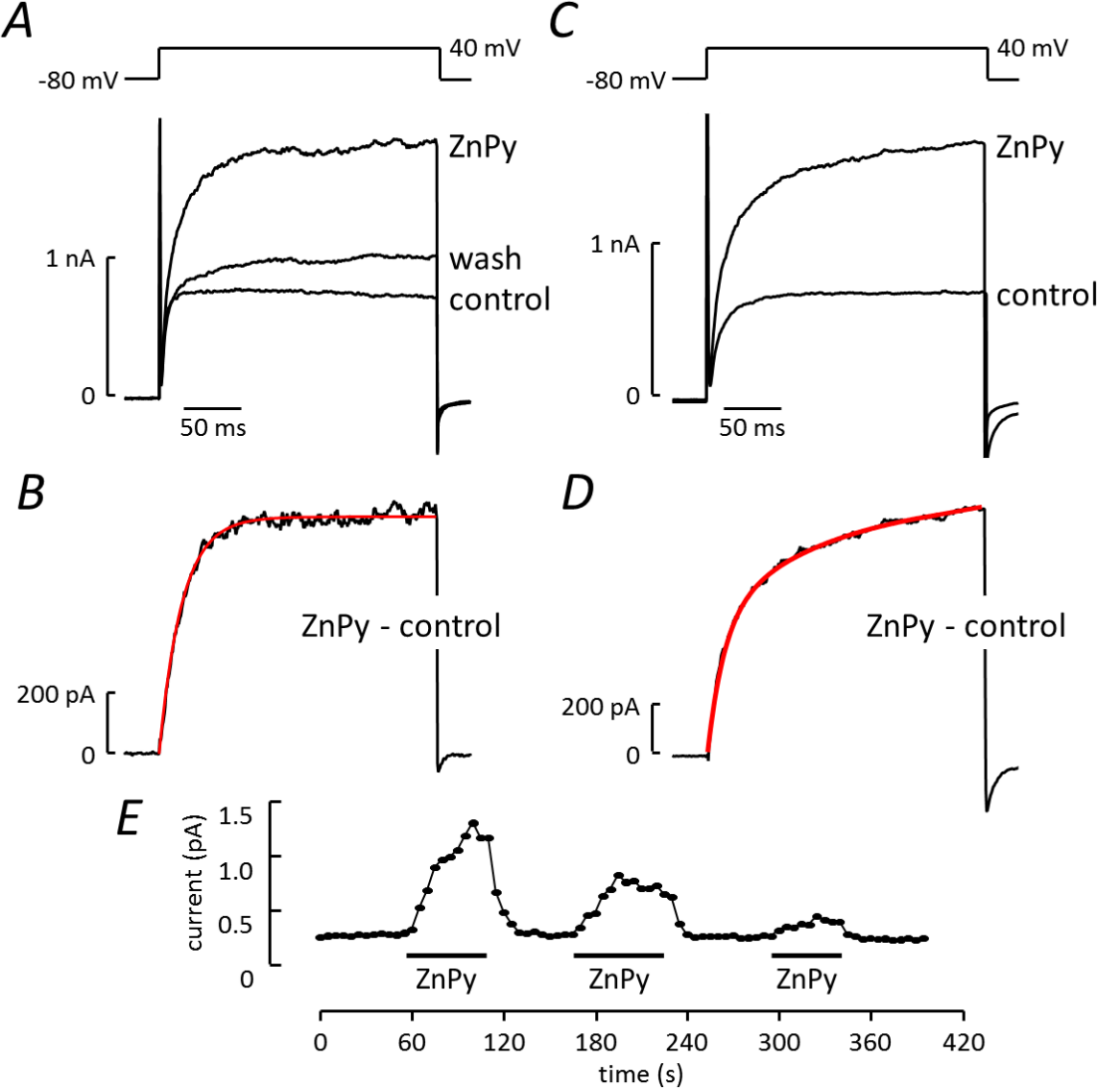
Properties of the ZnPy-induced current. ***A*,*C*** Examples of currents activated by a voltage step from -80 to 40 mV under control conditions, in the presence of 10μM ZnPy and after washing. Voltage protocol above traces. ***B*.** Difference current generated from the traces in ***A*** by digital subtraction of the control record from the record obtained in the presence of ZnPy. The best fit exponential function (red) with τ = 19.6ms is superimposed on the activation phase. ***D*.** Difference current generated from the traces in ***C***, with the exponential function that best fit the activation time course (red) superimposed on the trace: two components were required (τ_1_ = 13.2ms, τ_2_ = 101ms), the faster component contributing 50.6% of the current. ***E*.** Plot of current amplitude against time with ZnPy applied at the times indicated by the bars.

**Fig 5:**
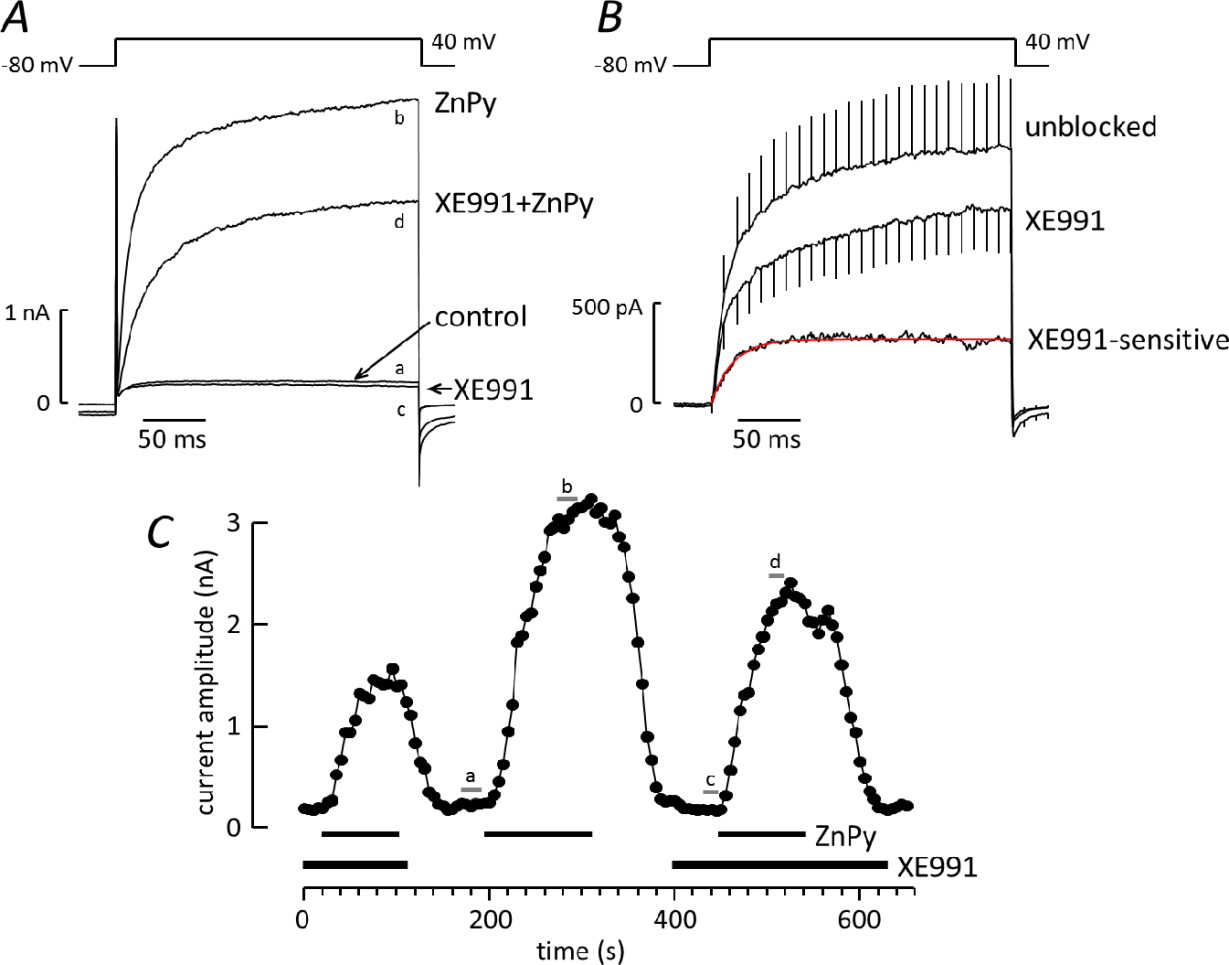
XE991 reduces the ZnPy-induced current. ***A***. Records of current activated by a voltage step from -80 to 40mV before and after exposure to 10μM ZnPy, under control conditions or in the presence of 10μM XE991. Each trace is the average of 3–4 consecutive records. ***B*.** Currents activated by ZnPy: traces are the average of records from 7 cells with bars indicating SEM. The ZnPy-induced current was obtained by subtracting control records from those in the presence of ZnPy (unblocked), or records in the presence of XE991 from those in the presence of XE991 plus ZnPy. The XE991-sensitive component was obtained by subtracting the ZnPy-induced current recorded in the presence of XE991 from that in its absence. The activation phase of the XE991-sensitive current was best fit by an exponential function (red), superimposed on the trace, with τ = 17.8ms. ***C*.** Plot of current amplitude against time, with ZnPy and XE991 applied as indictaed by the bars. Letters indicate amplitudes of traces in panel ***A***.

The effect of ZnPy was further investigated on currents activated by a voltage step from -80mV to 40mV, applied at 5s intervals (Fig 4). Under control conditions, ZnPy increased current amplitude from 532 ± 84 pA to 2 ± 0.3 nA (n = 33). ZnPy-induced current was isolated by subtracting control records from those in the presence of ZnPy (Fig 4). In one third of cells (10 out of 30) the Zn-Py-induced current activated exponentially following the voltage step (Fig 4A and 4B), with mean time constant (τ) = 64ms (33-95ms). In the remaining cells (Fig 4B and 4D), a double exponential function was required to fit activation, with mean time constants τ_1_ = 13 (9–16) ms and τ_2_ = 112ms (75-149ms). On average, the fast component accounted for 53±4% of the current. As illustrated in Fig 4E, the maximum ZnPy effect was reached in around 2 min and current usually returned to baseline within a few minutes of washing. Repeated ZnPy applications, separated by 4 min wash periods, reproducibly increased current amplitude, although in some cells the magnitude of the response decreased over time (Fig 4E) while in others it increased (e.g. Fig 5C).

Fig 5 illustrates the effect of XE991 (10 μM) on the voltage-activated K^+^ current at 40mV, in the absence and presence of ZnPy. XE991 reduced the current at 40mV by 19 ± 3% (n = 18) from 676 ± 78 to 550 ± 66 pA. In the presence of XE991, ZnPy retained its ability to enhance the current, albeit with reduced effect. To account for time-dependent changes in the ZnPy-induced current, the effect of XE991 was quantified by comparing the amplitude of the current evoked in the presence of XE991 to the average of responses before XE991 exposure and following its removal, or from the average of two responses in XE991 that flanked a control (as in Fig 5C). This indicated a significant inhibition of the ZnPy current by XE991, amounting to 33±10% (n = 7, one sample t-test). Subtracting the current evoked by ZnPy in the presence of XE from that in its absence revealed the XE991-sensitive component: Fig 5B shows the average from 7 cells. Current activation followed an exponential time course with mean τ = 17ms (16.3–17.7ms). Inhibition by XE991 was apparent within 1–2 min and was reversible (Fig 5C).

**Fig 6:**
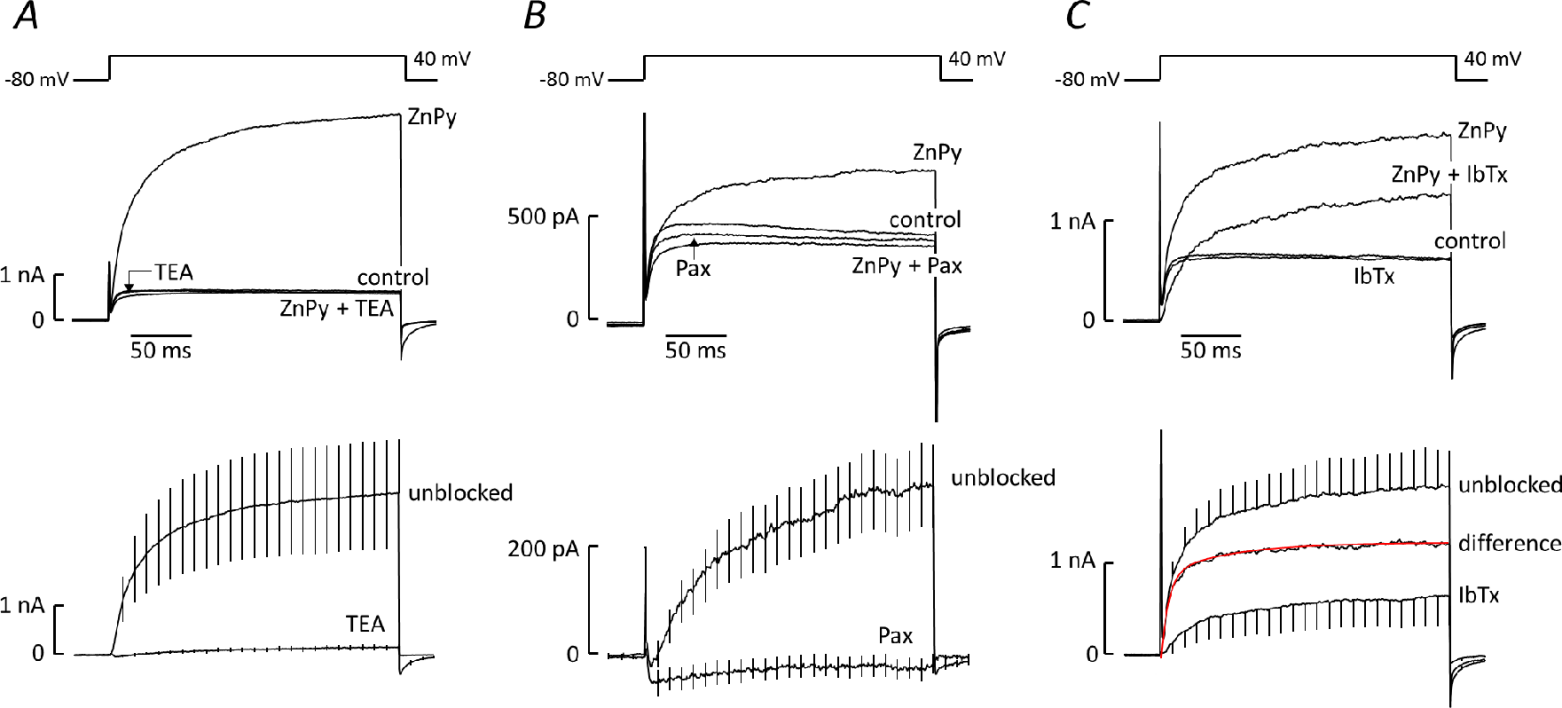
Effects of BK_Ca_ channel blockers on the ZnPy-induced current. Upper traces show currents activated by a voltage step from -80 to 40mV before and after exposure to 10μM ZnPy, under control conditions or in the presence of 10mM TEA ***(A)***, 1μM paxilline (Pax, ***B***) or 50nM iberiotoxin (IbTx, ***C***). Each trace is the average of records from 6 (TEA and iberiotoxin) or 8 (paxilline) cells. Lower panels show the ZnPy-induced currents, obtained by digitally subtracting records in the absence of ZnPy from those in its presence, either in control conditions (unblocked) or after exposure to a BK_Ca_ blocker. The iberiotoxin-sensitive component in ***C***, shown as the difference between the unblocked and iberiotoxin-inhibited currents, is superimposed by a double exponential function (red) that best fits the activation time course, with τ_1_ = 6.2ms and τ_2_ = 48ms: the fast component contributed 75.3% of the current.

BK_Ca_ channel blockers inhibited the voltage-gated current at 40mV to the same extent as previously reported [19]. TEA (10mM) reduced current amplitude by 18 ± 3% (n = 6) from 648 ± 205 to 543 ±183 pA. Paxilline (1μM) reduced it by 16 ± 4% (n = 9) from 433 ± 109 to 354 ±81 pA. Iberiotoxin (50 nM) caused an 11 ± 3% (n = 9) reduction from 520 ± 134 to 477 ± 134 pA. BK_Ca_ channel blockers also suppressed the ZnPy-induced current. Both TEA (Fig 6A) and paxilline (Fig 6B) essentially abolished the response to ZnPy, inhibition amounting to 97±2% (n = 5) and 112±7% (n = 7), respectively. Iberiotoxin (50nM) was less effective (Fig 6C), reducing the ZnPy-induced current by 40±9% (n = 5, differs from 0%, one-sample t-test). The iberiotoxin-sensitive component of the ZnPy-induced current, determined from the difference between the currents evoked in the presence and absence of the toxin (Fig 6C), activated bi-exponentially, the faster component accounting for 75% of the current. From fits to individual records the mean time constants were τ_f_ = 6.3ms (5.9–6.6ms) and τ_s_ = 57ms (52-63ms).

### Zinc pyrithione-induced current at 0 mV

**Fig 7:**
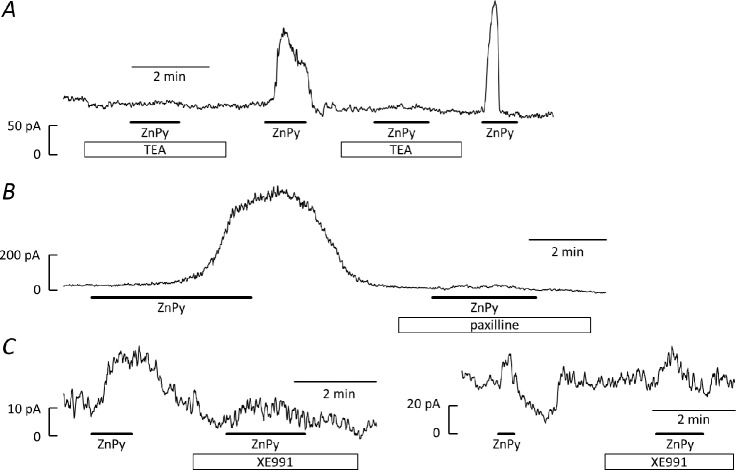
Effect of ZnPy on the residual current recorded at 0 mV. Typical traces showing the current remaining after clamping a cell at 0 mV for ≥5 min, followed by the application of ZnPy (10μM) on its own or in the presence of 10mM TEA ***(A)***, 1μM paxilline ***(B)*** or 10μM XE991 ***(C)***. Drugs applied as indicated by the bars.

To directly observe the effect of ZnPy on current, the membrane potential was clamped at 0 mV for ≥5 min [21]. This protocol activated outward current, which then declined by 76±2% (n = 22) as voltage-gated K^+^ channels inactivated, leaving a residual current of 39±5 pA (n = 36). The subsequent application of ZnPy (10μM) evoked additional outward current in 32 of 36 cells, which returned to baseline after washing. As illustrated in Fig 7, the amplitude of the response varied widely. While occasional cells responded to ZnPy with nA currents (Fig 7B), <100pA was evoked in 80% of cells (median = 26pA, mean = 253±87 pA, n = 36). TEA (10 mM) and paxilline (1μM) essentially abolished the ZnPy-induced current (Fig 7A and 7B), reducing it by 96±3% (n = 6) and 82±8% (n = 9), respectively. XE991 did not prevent ZnPy from evoking current, but may have reduced it (Fig 6C). Unfortunately, the variability in ZnPy responses, both between cells and between applications to the same cell, combined with difficulty in obtaining sufficiently long recordings for multiple ZnPy applications, made the effects of XE991 difficult to quantify. Exposure to XE991 appeared to reduce the response to ZnPy in 4 cells, but potentiated it in a fifth, giving an average reduction of 33±27% (n = 5), which did not reach statistical significance (Wilcoxon Signed Rank Test). Power analysis indicates that measurements on >30 cells would be required to detect 30% inhibition with 80% power and p<0.05, which is not practical.

## Discussion

ZnPy relaxed pulmonary arteries with IC_50_ of 4μM and was therefore more potent than retigabine or flupirtine [4]. As it relaxed arteries constricted by blocking Kv7 channels with XE991, its mechanism must not be restricted to the activation of Kv7 channels. ZnPy activated voltage-dependent K^+^ current and evoked membrane hyperpolarization in PASMC. The Kv7 channel blocker, XE991, prevented ZnPy from hyperpolarising the membrane and reduced its effect on current, consistent with the involvement of Kv7 channels. TEA and paxilline essentially abolished both the hyperpolarization and K^+^ current evoked by ZnPy, whereas iberiotoxin had no effect on the membrane potential response but reduced the current. The results are consistent with ZnPy activating two components of current that differed in their activation kinetics and pharmacology. The XE991, TEA and paxilline sensitive current most likely resulted from Kv7 channel activation, whereas BK_Ca_ channels can account for the component inhibited by TEA, paxilline and iberiotoxin. The inability of iberiotoxin to prevent the ZnPy-induced hyperpolarisation implies that it resulted from the Kv7 component.

ZnPy activates Kv7 channels with EC_50_ values in the low micromolar range [12,13,22,23]. Thus at 10μM ZnPy, Kv7 channels in PASMCs should be near maximally activated. In PASMCs, ZnPy activated current across a wide range of membrane potentials. This distinguishes it from retigabine, which produced detectable current only at negative membrane potentials [4] Although ~10% of cells did not hyperpolarise in response to ZnPy, this is a lower failure rate than seen with retigabine, which failed to hyperpolarise nearly 30% of PASMCs [4]. This difference could reflect the activation of different Kv7 subunits by the two drugs, or the ability of ZnPy, but not retigabine, to increase maximal conductance [12]. The greater efficacy of ZnPy on K^+^ current also resulted from a lack of selectivity for Kv7 channels, as indicated by the ability of iberiotoxin to inhibit ZnPy-induced current, but not hyperpolarisation. Kv7 channels are sensitive to inhibition by TEA, with most isoforms being substantially blocked at 10mM [24–26]. The TEA sensitivity of the ZnPy-induced current and hyperpolarization is therefore consistent with Kv7 channel involvement. Nevertheless, at that concentration, TEA also blocks BK_Ca_ channels and its effects on current were likely due to the combined block of both types of channel.

Paxilline is widely used as a potent (IC_50_<10nM) and selective BK_Ca_ inhibitor and had little effect on voltage-dependent K^+^ currents in several cell types [27]. The ability of paxilline to abolish responses to ZnPy in PASMCs, especially in comparison to the highly specific BK_Ca_ blocker, iberiotoxin [28], is therefore a surprise. At micromolar concentrations, paxilline does however inhibit Ca^2+^-activated Cl^-^ channels [29], inositol 1,4,5-trisphosphate receptors [30] and the sarco/endoplasmic reticulum Ca^2+^-ATPase (SERCA) [31], as well as stimulating glioma cell apoptosis independently of BK_Ca_ or SERCA inhibition [32]. There are no reports of tests for paxilline inhibition of Kv7 channels, but it may represent an additional off target effect of the drug at μM concentrations. Since neither TEA nor paxilline mimicked the depolarising effect of XE991 on the resting membrane potential, the Kv7 channel open at rest and mediating the depolarising effect of XE991 must be distinct from the Kv7 channels activated by ZnPy to evoke hyperpolarisation.

A large portion of the ZnPy-activated current (40%) was inhibited by all three BK_Ca_ channel blockers, TEA, paxilline and iberiotoxin, and was therefore mediated by BK_Ca_ channels. Despite their contribution to current, BK_Ca_ channels did not mediate the hyperpolarization response to ZnPy, because iberiotoxin did not prevent it. Voltage and Ca^2+^ act synergistically to open BK_Ca_ channels [33]. With millimolar concentrations of EGTA (in the pipette solution) to buffer the intracellular Ca^2+^ concentration ([Ca^2+^]_i_) and prevent Ca^2+^ from interacting with the channels, large BK_Ca_ currents can still be detected at positive potentials, despite not influencing the resting membrane potential [18,34]. The activation of current by ZnPy was voltage-dependent, so despite the large enhancement of current at positive potentials its effects on BK_Ca_ at the resting potential appear to be insufficient to change it. In constricted arteries, where [Ca^2+^]_i_ is expected to rise to μM levels and shift the activation of BK_Ca_ channels to negative potentials [33], enhancement of the current by ZnPy could become important. BK_Ca_-mediated hyperpolarisation in these conditions could account for the ability of ZnPy to relax arteries constricted by depolarisation induced by XE991.

The enhancement of K^+^ current at 40mV by ZnPy was of variable amplitude. Clamping cells at 0mV removed a large portion of the voltage-gated K^+^ current by inactivation. Unfortunately, in these conditions the ZnPy response appeared even more variable and block by XE991 could not be assessed with confidence. There are several potential sources of this variability. Firstly, 0mV is on the cusp of the current-voltage relationship, so current is more sensitive to variation. Secondly, the contributions of BK_Ca_ and different voltage-gated K^+^ channels vary widely among PASMCs [35–38]. This probably explains why the ZnPy current activated exponentially in response to a voltage step in some cells, but followed a bi-exponential time course in others. Whilst it would be ideal to study ZnPy at the resting potential, the small amplitude of currents in physiological conditions precludes this. According to Ohm’s Law and the average input resistance (4GΩ) measured in these experiments, ZnPy would need to add only 2.5pA of current to evoke 10mV hyperpolarisation. As PASMC often have input resistances >10GΩ [20,21], even smaller currents could be effective.

To conclude, ZnPy hyperpolarises PASMC by activating XE991, TEA and paxilline-sensitive K^+^ channels, most likely Kv7 channels. ZnPy also activates BK_Ca_ channels, but they do not mediate its effect on the resting membrane potential when [Ca^2+^]_i_ is low. This lack of ZnPy selectivity limits its usefulness for studying Kv7 channel function in intact arteries.
